# Skip Segment Hirschsprung Disease Managed by Pull-Through of the Right Colon

**DOI:** 10.1055/s-0041-1726347

**Published:** 2021-03-25

**Authors:** Hira Ahmad, Alejandra Vilanova-Sánchez, Isabel Amengual, Laura Guerra-Pastrian, Marta Garrido-Pontnou, Cristina Montalvo, Alba Bueno, Jacob Langer, Richard J. Wood, Marc A. Levitt

**Affiliations:** 1 Department of Pediatric Colorectal and Pelvic Reconstructive Surgery, Nationwide Children's Hospital Columbus, Ohio, United States; 2 Colorectal and Urogenital Reconstruction Unit, La Paz University Hospital, Madrid, Spain; 3 Department of Pathology, Son Espases University Hospital, Palma de Mallorca, Illes Balears, Spain; 4 Department of Pathology, La Paz University Hospital, Madrid, Madrid, Spain; 5 Department of Pathology, Vall d'Hebron Hospital Universitari, Barcelona, Catalunya, Spain; 6 Department of Pediatric Surgery, Son Espases University Hospital, Palma de Mallorca, Illes Balears, Spain; 7 Department of Pediatric Surgery, Hospital Universitario La Paz, Paseo de la Castellana 261, Madrid, Madrid, Spain; 8 Division of General and Thoracic Surgery, SickKids, Toronto, Ontario, Canada; 9 Division of Colorectal and Pelvic Reconstructive Surgery, Children's National Medical Center, Washington, District of Columbia, United States

**Keywords:** calretinin stain, reoperation, enterocolitis, continence, aganglionosis

## Abstract

Hirschsprung disease is the most common neurocristopathy in children, resulting in the congenital loss of enteric ganglia. Rare reports of skip lesions have previously been reported in the literature. We present a case of skip lesions known prior to surgery and managed by pull-through of the right colon that allowed the preservation of the colon.

## Introduction

Skip lesions have rarely been reported in patients with Hirschsprung disease (HD) especially before the definitive pull-through procedure. We present a case of an infant with HD with skip lesions known prior to their pull-through and managed with a pull-through of the right colon.

## Case Presentation

**Fig. 1:**
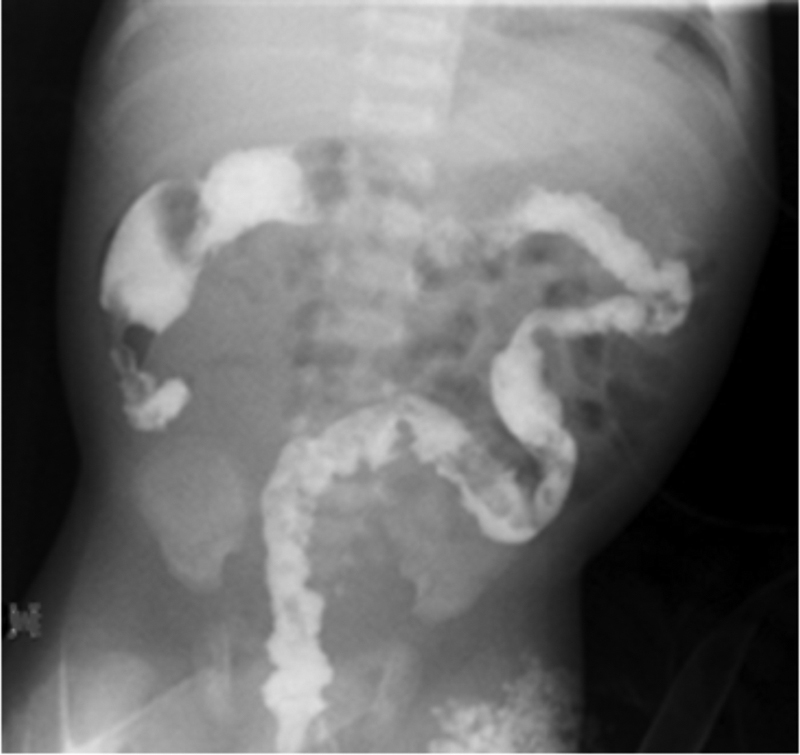
Repeat contrast enema concerning for long-segment Hirschsprung disease: microcolon, the question mark-shaped colon, and the lack of features in an otherwise normal colon.

A full-term baby boy who had failed to pass meconium at 48 hours had persistent abdominal distention and bilious emesis. An upper gastrointestinal contrast study was normal but when contrast passed to the colon a concern for inspissated stool was entertained. Due to persistent distention, he underwent exploratory laparotomy with enterotomy (ileostomy, 24 cm from ileocecal valve) and terminal ileal washout. The baby was operated on for presumed intestinal obstruction and no contrast enema was done due to significant abdominal distention and to prevent delaying the surgery. At the time of surgery, full-thickness sigmoid colon biopsies were obtained, with intraoperative frozen section reporting presence of ganglion cells, but the tissue review on permanent was deemed by the pathologists to be inadequate and no ganglion cells were seen. He initially did well and was tolerating oral exclusively formula intake with about five spontaneous bowel movements per day. Cystic fibrosis testing, both metabolic and genetic, was negative. He was discharged home 28 days later. Four days after discharge, he presented again with obstructive symptoms. He underwent a contrast enema that was suspicious for long segment HD, microcolon, and the question mark-shaped colon ( Fig. 1 ). Anorectal manometry showed an absent rectoanal inhibitory reflex (RAIR). Since at patient's 22 weeks prenatal ultrasound, a left pelvic kidney with double ureteral system was noted, a pelvic ultrasound was obtained. A pelvic ultrasound showed hydronephrosis in a left pelvic kidney and a dimercaptosuccinic acid scan showed it had no function; the right kidney was normal. A voiding cystourethrogram showed no vesicoureteral reflux.

**Fig. 2:**
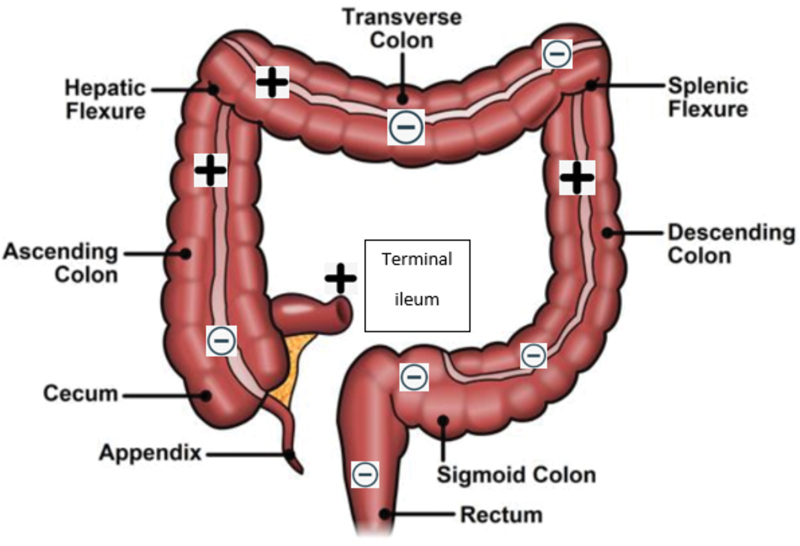
Results of colonic mapping at the time of ileostomy creation, showing Hirschsprung disease with skip lesions. (+) denotes presence of ganglion cells. (–) denotes absence of ganglion cells.

Rectal biopsy showed absence of ganglion cells in the submucosa, and loss of calretinin expression in the lamina propria of the mucosa and no hypertrophic nerves. He underwent a diverting ileostomy with colonic mapping with findings shown in Fig. 2 . Notably, he had no ganglion cells in his cecum but had ganglion cells in the ascending colon and hepatic flexure. The transverse colon and sigmoid were aganglionic. The left colon had ganglion cells. This pathology was reviewed by two different pathology departments and both agreed with the findings. This patient was then referred to an HD referral center for another opinion, and the entire complete set of slides (966 sections) from the colonic mapping done in the first hospital were reviewed by a third pathologist. After extensive discussion with the parents, the patient was taken to the operating room for repeat colonic mapping as there was concern for the possibility that the slides had been mislabeled. We repeated the mapping and confirmed that the findings as originally determined were accurate ( Fig. 2 ).

**Fig. 3:**
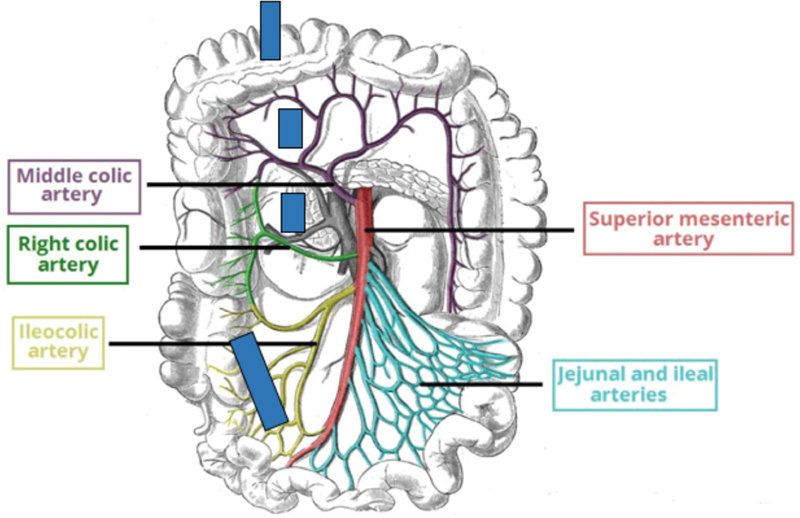
Colonic vasculature and our plan for the resection and pull-through. The blue lines mark the vasculature that will be taken down for the resection, derotation, and eventual pull-through of the right colon.

**Fig. 4:**
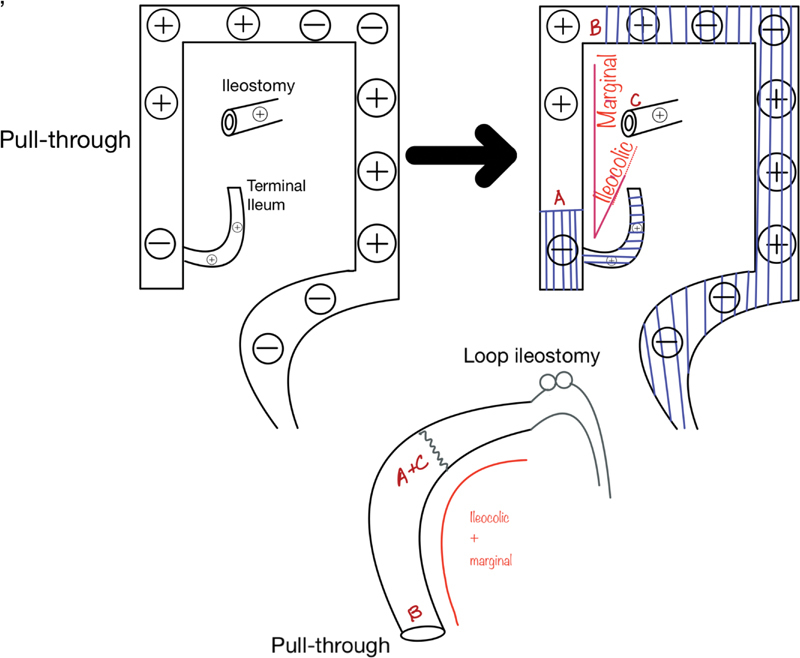
Cecal resection, extended left colectomy, ileocolic anastomosis, right colonic derotation pull-through of the right colon. The last figure shows the final postoperative anatomy.

With this ganglionic map in mind, we performed a transanal proctectomy, with transabdominal (via laparotomy) excision of sigmoid, left colon, and transverse colon. The middle colic vessels were divided, carefully preserving the right marginal vessels from the ileocolic vessels to the point of good ganglion cells at the hepatic flexure with the idea to preserve the ganglionic right colon (8 cm). Intraoperative biopsies from hepatic flexure and cecum were done and ganglion cells were identified only in myenteric plexus from hepatic flexure. We then performed a cecal resection, carefully preserving the blood supply to the right marginal vessels from the ileocolic vessels. An ileocolic anastomosis was performed (ileum to right colon with cecum now removed) and the right colon was then derotated to perform the pull-through down the right pelvis ( Figs. 3 and 4 ). Derotation has been described in adult literature extensively as Deloyers procedure. It is safe and associated with good long-term results. 1 The diverting ileostomy was left in place. The patient underwent examination under anesthesia 4 weeks postoperatively that showed a well-healed anastomosis and no stricture. The parents were instructed to pass stool from the ileostomy into the distal segment daily, and he was able to successfully control the stool out of his neorectum. He then underwent ileostomy takedown 4 months later. On his 1 year follow-up (he is currently 2 years of age), the patient is stooling normally with Bristol stool chart type 4 and 5, 4 to 5 stools per day.

**Fig. 5:**
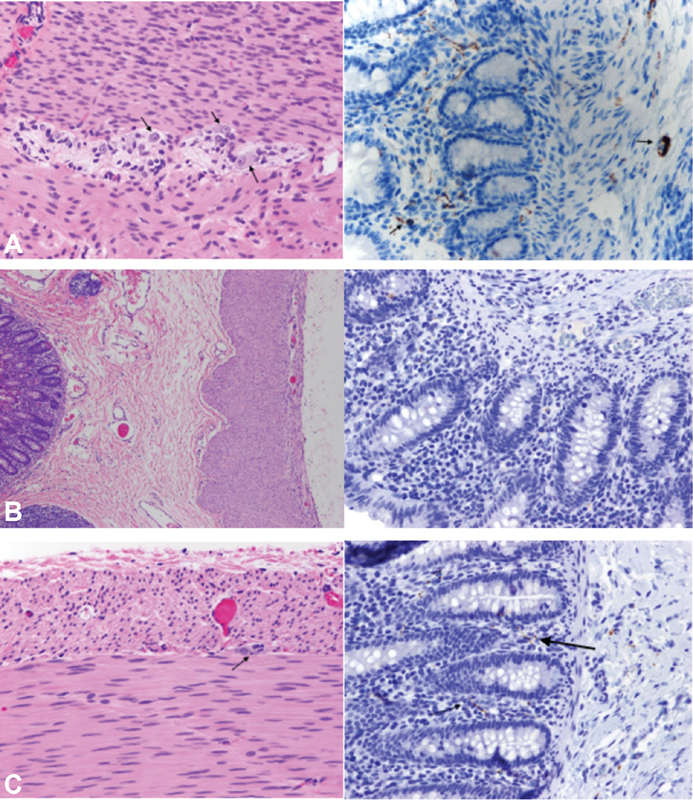
( **A** ) Section of normal ileum (proximal margin) with ganglion cells in the myenteric plexus (hematoxylin and eosin [H&E] left, marked by arrows) and normal expression of calretinin in lamina propria of the mucosa (right, marked by arrows). ( **B** ) Section of aganglionic segment of the cecum demonstrating absence of ganglion cells in the submucosal and myenteric plexuses (H&E left) and loss of calretinin expression in lamina propria of the mucosa (right). ( **C** ) Descending colon with isolated ganglion cell in the myenteric plexus (H&E left marked by arrows) and few nerve fibers in lamina propria stained with calretinin (right, marked by arrows)

In the pathological study of the surgical specimen, the proximal margin (ileum) was normal, with presence of ganglion cells in the submucosal and myenteric plexuses and normal expression of calretinin in the mucosa. In the cecum, transverse colon, splenic flexure, sigmoid colon, and rectum there were no ganglion cells in the plexuses and there was loss of calretinin expression in the mucosa. In the ascending colon, hepatic flexure, and descending colon, scattered ganglion cells were identified in both plexuses, with focal expression of calretinin in the mucosa. The overall appearance of these “skip areas” resembled “transition zones” and never showed a normal number or distribution of ganglion cells ( Figs. 5A–C ).

## Discussion

HD is a congenital intestinal obstruction due to distal aganglionosis, defined by the lack of ganglion cells in the myenteric and submucosal plexuses of the distal intestine to the level of proximal ganglionic bowel. It is seen in 1 in 5,000 live births and its management involves resection of the aganglionic bowel and pull-through of normal bowel to restore functional continuity. 2 3 All definitive Hirschsprung operations serve this vital purpose, no matter which type of procedure is selected.

Segmental aganglionosis (skip lesions) is an extremely rare entity in HD, and in fact many doubt its existence. The first reported case of a skip HD lesion was published in 1954. 4 Skip segment HD refers to normally ganglionated intestine, surrounded proximally and distally by aganglionosis. 5 On the other hand, in zonal aganglionosis, an aganglionic segment is sandwiched or interposed between segments of normally innervated or ganglionated bowel. 6 7 Some of these cases used the appendix to show skip lesions but this is an incorrect concept as many appendices are aganglionic. 8

The pathology of typical HD is sustained contraction of the aganglionated bowel segment due to absence of ganglion cells of the myenteric and submucosal plexuses. The lack of ganglion cells, derived from neural crest cells, is usually due to loss of coordination of the survival, migration, proliferation, and/or differentiation of the progenitor cells in the gastrointestinal (GI) tract. Generally, this occurs either due to arrest in migration of neural crest cells to reach their final GI destination 9 or due to failure of proliferation, survival or differentiation after the migration has already occurred. 10 Neither of these hypotheses explains the skip lesion concept that has led to skepticism of its existence. Even though there has been a relative paucity of data in reporting skip lesions, a review identified 23 reported cases in the literature. 11 One theory to explain the skip lesions is that neuroblasts, when migrating in a craniocaudal direction during neural crest migration, cross the mesenteric border to a more distal part of the intestine and as a result end up well ahead of the wavefront, colonizing an area within the aganglionic segment. 12 13

Most cases of skip lesion HD are total colonic aganglionisis (TCA) reported to be 92%, and 8% were rectosigmoid HD. Of the TCA cases, 41% had a skip segment in the transverse colon, 27% in the ascending colon, 9% in the cecum, and 23% had multiple skip segments. 11 Skip segment thus should be suspected in patients with TCA. In cases with TCA, colonic mapping should be performed. In these cases, biopsies are performed in the rectum, sigmoid colon, descending colon, splenic flexure, transverse colon, hepatic flexure, ascending colon, and the cecum. 14 Patients with skip lesion HD are described to present with failure to pass meconium, chronic constipation, bilious emesis, and abdominal pain. Diagnosis of HD is made in most patients using suction rectal biopsy findings of aganglionosis, and hypertrophic nerve trunks in the submucosa. 15 Calretinin immunohistochemistry can be used as an adjunctive method to diagnose HD, because of the loss of the staining pattern of small nerve fibers of the lamina propria, muscularis mucosae, and submucosa of aganglionic zones. 16 We perform anorectal manometry in patients over 1 years old for an evaluation of constipation in which Hirschsprung's is a possibility or internal sphincter achalasia. Typically, if the manometry shows an absent RAIR, we perform a rectal biopsy at the time. We prefer to perform manometry awake; however, this is not always possible. The RAIR can be determined even in an anesthetized patient.

It is pertinent to understand the vascular anatomy of the colon especially in patients with skip lesion HD to allow reconstruction with maximum colonic salvage. Resection of the colon is based on the arterial supply to its various anatomic divisions ( Fig. 3 ). The superior mesenteric artery, via ileocolic and right colic artery, supplies the cecum and the ascending colon respectively. These arteries then further give off vasa recta that form arcades adjacent to the colonic wall. We took advantage of the colonic anatomy and resected the cecum only by dividing the vasa recta, and preserved the marginal artery from ileocolic artery to supply the eventual pull-through segment and allowed preservation of the ganglionated right colon (8 cm).

It is possible that many cases of skip lesion HD have been missed and dismissed by surgeons and pathologists over the years because the idea contradicts the well accepted theory of neural crest migration in the gut, and the disturbance of migration in HD. This condition has divided pediatric surgeons, with many only believing this phenomenon after experiencing this condition in their own patients. We can confirm with this report that skip lesion HD does exist.

Skip lesion HD is an extremely rare entity in HD. If known prior to the surgical intervention, careful preoperative planning may help preserve functional colon in patients with this unique condition.

## Notes

New Insights and the Importance for the Pediatric Surgeon Pediatric surgeons should have a high index of suspicion for skip lesions especially in the presence of total colonic aganglionosis.
